# Construction and application of an ICU nursing electronic medical record quality control system in a Chinese tertiary hospital: a prospective controlled trial

**DOI:** 10.1186/s12912-024-02178-3

**Published:** 2024-07-18

**Authors:** Shuai Zhang, Yin Yin Quan, Juanhong Chen

**Affiliations:** Emergency and Critical Care Center, Intensive Care Unit, Zhejiang Provincial People’s Hospital (Affiliated People’s Hospital, Hangzhou Medical College), Hangzhou, Zhejiang 310014 China

**Keywords:** Intensive care units, Digitalization of nursing, Electronic medical record, Nursing quality control

## Abstract

**Background:**

ICU nurses provide critical care and meticulously document electronic medical records (EMRs), tracking vital signs, interventions, and medication hourly. Despite China’s ICUs effectively integrating real-time monitor and ventilator data into EMRs, challenges persist. Patient movements can introduce inaccuracies, and the demands of critical care may lead nurses to miss assessments like pain and nutrition. Traditional manual EMR verification is inefficient and error-prone, highlighting the urgent need for standardized, technology-aided EMR practices in ICU nursing.

**Objective:**

This study aimed to describe the development and evaluation of an electronic medical records quality control system implemented in a Chinese tertiary care ICU setting, where current practices impact the accuracy of electronic medical records.

**Methods:**

A prospective controlled trial was conducted with 600 ICU patients in Zhejiang Province from January to December 2023. An automated EMR quality control system was implemented in July 2023, facilitating real-time data collection and quality control for vital signs, medication management, and nursing evaluations.

**Results:**

After implementing the ICU nursing electronic medical record quality control system, the prevalence of false data on vital signs decreased from 9 to 1.33%. Additionally, the incidence of incomplete medication administration dropped from 3.33 to 1.67%, and the rate of missing evaluations of assessment items in EMRs was reduced from 8 to 1.33%. Besides, the average time spent on quality control of the electronic medical records was 62 (48,76) seconds per record, which was significantly lower than the 264 (195.5,337.5) seconds using the traditional method. The nurses’ satisfaction with the nursing electronic medical record quality control was (105.73 ± 9.31).

**Conclusions:**

The ICU nursing electronic medical record quality control system has led to substantial improvements in the quality and reliability of EMRs. The reduction in false data on vital signs, instances of incomplete medication administration, and missing evaluations of assessment items demonstrates the system’s positive impact on nursing documentation practices. These improvements not only enhance the accuracy of patient records but also contribute to better patient care and safety within the ICU setting.

## Introduction

Following the development of medical informatization, electronic medical records (EMRs) have gained popularity [1]. The nursing electronic medical record is an essential part of the hospital information system, and its quality reflects the nursing quality and management level of medical institutions. EMRs and electronic clinical nursing documentation enhance the quality and completeness of documentation and promote a patient-centered approach compared to manual nursing documentation [2]. Electronic medical records facilitate improvements in patient care [3]. The National Health Commission of the People’s Republic of China released the “Grading Evaluation Standards for the Application Level of Electronic Medical Record Systems” in December 2018. The six-level electronic medical record standard mentioned that each medical service project should enable data collection, recording, and sharing. Moreover, the entire process status should be displayed, providing real-time data verification, prompts, and control functions [4].

In addition, the National Health Commission issued the Action Plan for Further Improving Nursing Services (2023–2025) in June 2023, which mentioned that the quality of nursing should be improved by using information technology to reduce unnecessary writing. This would enable nurses to have more time to provide direct nursing care to patients [5]. In the intensive care unit (ICU), patients are continuously monitored, resulting in huge datasets [6]. A large and complex amount of data is recorded in the ICU’s EMRs, which undoubtedly increases the nursing workload [7]. Furthermore, a growing body of evidence suggests that EMRs integrated with ICU information systems lead to lower in-hospital mortality [8]. In addition to providing intensive nursing care to critically ill patients, ICU nurses are also responsible for documenting standardized electronic medical records. This includes recording vital signs, nursing interventions, medication administration, and pipeline assessments on an hourly basis. With the widespread adoption of information technology, ICU wards in China have successfully integrated monitor and ventilator data into electronic medical records, allowing for real-time capture of data throughout the entire duration of care. Currently, if the patient carries out actions such as coughing or rolling over, the vital signs column of the nursing electronic medical record may contain pseudo-data. Moreover, ICU nurses may overlook assessments of pain, nutritional tolerance, restraints, and other conditions while attending to critically ill patients.

At present, the quality control of nursing electronic medical records in many hospitals in China is still dominated by manual spot checks, and the automatic monitoring of electronic medical records has not yet been popularized [9]. The conventional quality control method for electronic medical records often involves manual verification, which is very time-consuming, prone to high omission rates, and subject to significant subjectivity. Our hospital introduced the hospital information system in March 2020 in accordance with the core requirements of the National Health Commission’s Level 6 electronic medical record application. The National Health Commission’s Level 6 electronic medical record assessment was successfully passed in July 2023. The monitor, ventilator, and ICU nursing electronic medical record system are connected by a wireless network. This allows for the real-time collection, transmission, and recording of the patient’s vital signs, thus minimizing errors associated with manual recording. Furthermore, ICU nurses can scan the barcode on the drug execution form using their personal digital assistant (PDA), enabling them to initiate, adjust, and terminate drug administration within the ICU nursing electronic medical record system. This implementation facilitates closed-loop drug management. Despite the convenience of the ICU nursing electronic medical record system, issues such as inaccurate data, delayed administration of medications, and incomplete evaluation management persist. As a result, a nurse was assigned to conduct daily quality control of ICU nursing electronic medical records. Therefore, this study successfully implemented an ICU nursing electronic medical record quality control system, aiming to provide ICU nurses with more time to focus on patient care and reduce the time spent on medical record documentation and quality control. The results of this implementation were found to be positive.

## Methods

The single-center, prospective controlled study was conducted in the ICU of a tertiary hospital in Zhejiang Province, China. The study included a sample of 600 patients who were admitted to the intensive care unit (ICU) of a tertiary hospital in Zhejiang Province between January and December 2023.

**Fig. 1:**
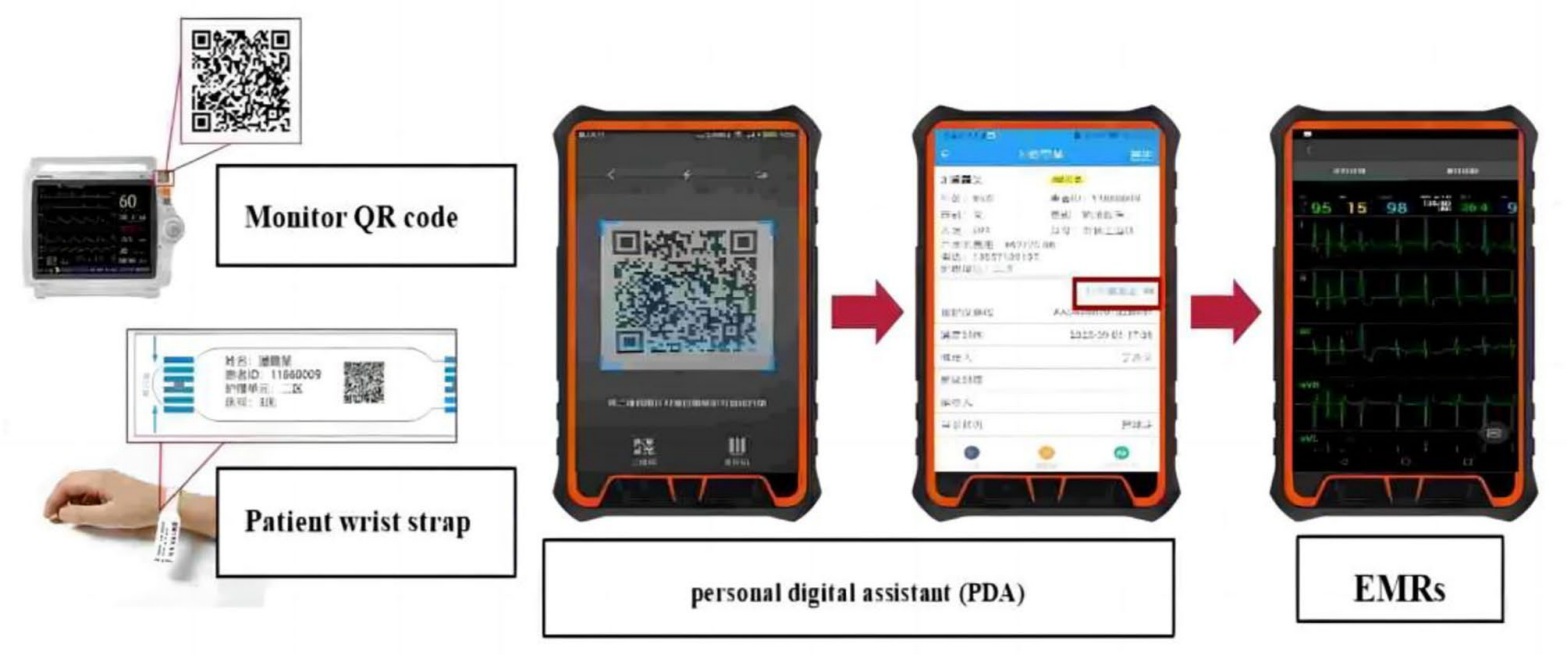
ICU nursing electronic medical record is linked with the monitor, real-time receiving patients’ vital signs data

**Fig. 2:**
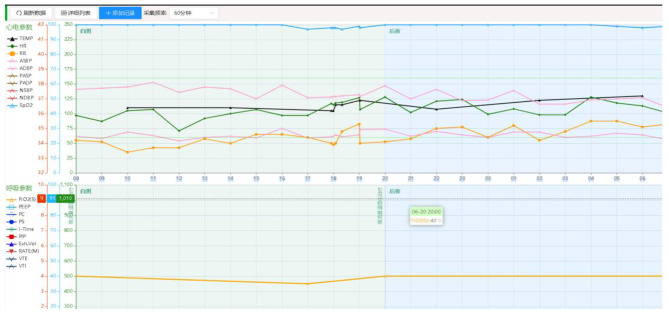
Color-coded Vital Sign Parameters in the Nursing Electronic Medical Record System

ICU nursing electronic medical records quality control (QC) was performed manually before June 2023. QC nurses performed periodical checks of the electronic medical records on a daily basis. To ensure the precision and uniformity of the EMR quality control process, our study explicitly defined ‘false data’ for the quality control nurses. ‘False data’ refers to any information within the recorded EMRs that deviates from the actual patient measurements or observations due to technical malfunctions or human entry errors. This encompasses, but is not limited to, erroneously documented vital signs, medication dosages, and nursing assessments. The quality control nurses were trained to identify and rectify these discrepancies using predefined criteria and real-time data validation against medical device outputs to ascertain data accuracy. As part of tertiary care requirements, nurses are required to record the patient’s vital signs, including monitor and ventilator parameters, every hour in the nursing electronic medical record. Patients with unstable conditions should be monitored more frequently. A nursing electronic medical record system wirelessly connected to monitors, ventilators, and other instruments was implemented in our ICU. This ensures that the patient’s vital signs are captured and recorded in the nursing electronic medical record (Fig. 1). For example, the electronic medical record system will collect vital signs from 1 AM to 12 PM for a mechanically ventilated patient. When there is a change in condition, vital signs can also be captured every minute, including heart rate, blood pressure, ventilator tidal volume, etc. The EMRs incorporates a color-coding mechanism to differentiate the documentation of vital sign parameters, thereby enhancing the readability and recognizability of the data (Fig. 2). Specifically, unique colors were assigned to each vital sign parameter as follows: Body Temperature is represented in black. Heart Rate is indicated in green. Respiratory Rate is denoted in orange. Blood Pressure readings are highlighted in pink. This color-coding system facilitates rapid identification and tracking of trends for each vital sign by the QC nurses. By visually inspecting the color-coded vital sign parameter curves, ICU QC nurses can identify pseudo-data and make artificial manual modifications. In terms of drug administration, our hospital has implemented closed-loop management of drugs. ICU nurses use a personal digital assistant (PDA) to scan quick response (QR) codes on drugs, presenting the patient information and scanned medication on the screen of the PDA. Nurses then input the medication administration dose and rate according to the prescription and click execute, which is recorded into the electronic medical system. When the medication ends, the QR code is scanned again to complete the medication administration in the ICU nursing electronic medical record. Nevertheless, a malfunction in the network or PDA may result in the medication not being properly terminated in the electronic medical record. Therefore, ICU QC nurses manually terminate the medication, creating a closed-loop medication management. In addition, ICU nurses are required to assess patients for physical restraints, medication sedation, mental status, and nutritional scores as required. Omissions are also added manually by the ICU QC nurse.

In this study, a research team was formed to investigate the quality control of electronic medical records (EMRs) in the Intensive Care Unit (ICU). The team consists of 3 members, including ICU nurse leader A with a senior professional title, nurse B with a master’s degree, and information engineer C. A is responsible for the content scheme construction of the nursing electronic medical record quality control system, B is responsible for reviewing literature and subsequent data collection and statistical analysis, and C is responsible for the construction of the system and technical maintenance during the use process. The team utilized the ICU nursing EMR system as a foundation and employed group brainstorming techniques to design quality control rules for the EMRs. Additionally, the team collaborated with information engineers to construct an ICU nursing EMR quality control system. This system enables real-time data collection and facilitates quality control measures for patient vital signs, medication management, and nursing evaluation. Furthermore, the system incorporates dynamic reminders and ensures the quality control of electronic medical records.

The ICU nursing electronic medical record quality control module mainly includes three parts: *vital signs*,* drug management*,* and evaluation management*. The vital signs section requires hourly records of the patient’s heart rate, oxygen saturation, systolic blood pressure, diastolic blood pressure, mean arterial pressure, and respiration. In addition, patients’ body temperature must be measured every 4 h, specifically at 6:00, 10:00, 14:00, 18:00, 20:00, and 24:00. Patients on mechanical ventilation require hourly measurements of vital signs, such as respiratory rate, minute ventilation, inhaled tidal volume, exhaled tidal volume, and oxygen concentration. If the above data is missing, the system reminds the nurse to supplement it in time. In addition, abnormal values in the data collected by the electronic medical record system, such as an exhaled tidal volume of < 150 ml, trigger an alarm. The nurse will make timely corrections according to the reminder to ensure the accuracy of the data. *The drug management* component of the system mandates strict adherence to physician-prescribed orders. The initiation and cessation of each medication administration are recorded, ensuring that medications are administered at the prescribed rate and within the specified time frame. If a nurse fails to document within the set timeframe, the system automatically generates a prompt to encourage immediate action, thereby facilitating closed-loop medication management. *Assessment management* includes the following: patient position and physical restraint should be assessed every 2 h; skin condition, level of consciousness, pupil size (left/right), upper limb muscle strength (left/right), lower limb muscle strength (left/right), breath sounds, sputum viscosity and volume, urine color, bowel sounds, dorsalis pedis artery pulse (left/right), lower limb edema (left/right), delirium assessment, high-risk pressure injury assessment, pain assessment, Glasgow Coma Scale, and enteral nutrition intolerance should be assessed every 4 h. If the nurse does not assess within the specified time, the system will pop up a reminder box. The reminder box disappears once the ICU nurse completes the assessment. Nurses are required to write up the nursing document hourly, in accordance with the standard of care for critically ill patients. Any deviation from this schedule is flagged for review, and the system issues a reminder to the responsible nurse to ensure timely documentation. The ICU nursing EMR quality control system was specifically designed to automatically detect and correct omissions in real-time, reducing the reliance on manual checks. The system operates on advanced algorithms capable of recognizing patterns and anomalies within the EMRs. It cross-references recorded data with predefined medical standards and real-time physiological monitoring outputs to identify discrepancies. Upon detection of an omission, the system triggers an automated alert and suggests the correct value based on the trending data and clinical context. This process occurs continuously and in real-time, ensuring that the EMRs are updated promptly without manual intervention.

The ICU nursing electronic medical record quality control system was implemented in July 2023. The time spent by nurses on medical record quality control before the system’s implementation (January to June 2023) was compared to after its implementation (July to December 2023). Additionally, the study examined the nurses’ satisfaction with the system, as well as the patient vital signs, medication management, and evaluation management in nursing EMRs.

### Data collection

In this research, a total of 300 nursing electronic medical records were randomly chosen to undergo manual quality control assessment before the implementation of the ICU nursing electronic medical record quality control system (January to June 2023). The manual quality control assessment team comprised five ICU nurses, all holding the position of head nurse. Each nurse was tasked with reviewing 10 different nursing electronic medical records every month, focusing on vital signs, medications, and assessment management sections from the previous 24 h. The time taken for each record inspection was recorded, from the start to the end of the inspection. Following the introduction of the ICU nursing electronic medical record quality control system (July to December 2023), another 300 nursing electronic medical records were randomly chosen for electronic quality control assessment. Similarly, each of the five nurses randomly selected 10 nursing electronic medical records for inspection every month. The quality control system prompted the nurses to review the nursing electronic medical records from the previous 24 h, and the time taken for each electronic medical record inspection was recorded. The inclusion criteria for the selected medical records were patients over 18 years old who had stayed in the ICU for longer than 24 h.

The study utilized the Clinical Nursing Information System Effectiveness Evaluation Scale, developed by Zhao [10], to assess nurses’ satisfaction with the ICU nursing electronic medical record quality control system. This scale comprises five dimensions: system quality, information quality, service quality, user satisfaction, and net benefits, including a total of 23 items. Each item was rated on a 5-point Likert scale, ranging from 1 (strongly disagree) to 5 (strongly agree). The total score ranges from 23 to 115, with a higher score indicating greater satisfaction with the management system. The original author granted authorization to use this scale in our study. In December 2023, the scale was given to the ICU nurses at the end of each shift and collected within 24 h. Following the scale integration into the questionnaire platform (www.wjx.cn) by the researchers, consent was sought from ICU nurses during their off-duty hours. They were requested to anonymously complete the questionnaire in a secluded environment. The reliability and validity of the scale in this context were confirmed through pilot testing prior to the full-scale implementation of the study.

### Statistical analysis

This study used R version 3.5.3 to analyze the data in this study. Means and standard deviations for metrics conforming to a normal distribution were analyzed using t-tests, while medians and quartiles were used for metrics following non-normal distributions. Analyses were conducted using the Wilcoxon rank-sum test, and counts were described by frequency and percentage using the *Chi-square* test. An analysis with a p-value of 0.05 was considered statistically significant.

In the statistical analysis of our study, we employed the Wilcoxon rank-sum test to compare the non-normally distributed continuous data between the two independent groups. Given that the data did not meet the assumptions of normality, as assessed by the Shapiro-Wilk test (*p* < 0.05), the Wilcoxon rank-sum test was selected for its robustness in evaluating differences without relying on the normality assumption. This test is particularly suitable for our dataset, as it is based on the ranks of the data rather than their actual values, making it a reliable choice for analyzing non-normally distributed continuous variables. The use of the Wilcoxon rank-sum test is indicated in Table 1, where the non-normally distributed continuous data are presented as M(P25, P75), reflecting the median and interquartile range, respectively.

## Results

Prior to and subsequent to the implementation of the ICU nursing EMR quality control system, 600 patient cases were analyzed to compare gender, disease diagnosis, and length of ICU stay. The results showed no statistically significant differences. However, a significant improvement in the EMR documentation was observed after the implementation of the ICU nursing electronic medical record quality control system, as shown in Table 1. Specifically, the prevalence of false data on vital signs decreased from 9 to 1.33%, indicating a more accurate reflection of patients’ conditions. Additionally, the incidence of incomplete medication administration dropped from 3.33 to 1.67%, suggesting improved compliance with medication protocols. Furthermore, the rate of missing evaluations of assessment items in EMRs was reduced from 8 to 1.33%, highlighting the system’s effectiveness in ensuring comprehensive nursing assessments.

**Table 1:** A comparison of the quality control time and content of ICU nursing electronic medical records before and after implementation.(*N* = 600)

Characteristics of patients		Before implementation (*n* = 300)	After implementation (*n* = 300)	Statistical values	*P*-value
Gender(%)	Female	130(43.33)	122(40.67)	0.438^a^	0.508
	Male	170(56.67)	178(59.33)		
Disease diagnoses(%)	Cardiac disease	208(69.33)	210(70.00)	2.498^a^	0.645
	Disease of the digestive system	28(9.33)	29(9.67)		
	Orthopedic disease	4(1.33)	8(2.66)		
	Disease of the Nervous system	19(6.33)	13(4.33)		
	Other diagnostic types of disease	41(13.67)	40(13.33)		
Length of stay in ICU[M(P25,P75),day]		5 (7, 10)	5 (7, 10)	18191.5^b^	0.623
The quality control time[M(P25,P75),second]		264(195.5, 337.5)	62(48, 76)	45,150^b^	<0.001
False data on vital signs in EMRs(%)	YES	27(9.00)	4(1.33)	261.231^a^	<0.001
	No	273(91.00)	296(98.67)		
Medication not administered to completion in EMRs(%)	YES	10(3.33)	5(1.67)	275.339^a^	<0.001
	No	290(96.67)	295(98.33)		
Missing evaluations of assessment items in EMRs(%)	YES	24(8.00)	4(1.33)	264.229^a^	<0.001
	No	276(92.00)	296(98.67)		

Note: EMRs: electronic medical records; a: Statistic value of the *Chi-square* test; b: Wilcoxon rank-sum test

In this research, a total of 48 satisfaction surveys were gathered to evaluate the quality control system of the ICU nursing EMRs. Four surveys were deemed invalid and excluded, resulting in 44 valid surveys. The recovery rate of valid surveys was 91.67%. Based on the data presented in Table 2, the ICU nurses expressed a relatively high level of satisfaction with this system, as indicated by an overall satisfaction score of (105.73 ± 9.31).

**Table 2:** The score of ICU nurse satisfaction scores for nursing EMR quality control system

Variable	Common nursing M(SD)
Total satisfaction	105.73(9.31)
system quality	18.23(2.05)
information quality	22.89(2.47)
service quality	18.25(2.07)
user satisfaction	23.61(1.91)
net benefits	22.75(2.60)

Note: EMRs: electronic medical records

## Discussion

ICU nurses are required to dedicate a greater amount of time and effort to patient care compared to those working in standard hospital wards, particularly for patients undergoing prolonged mechanical ventilation and suffering from infections. Studies (11–12) have indicated that nurse-patient interactions and the complexities involved in delivering care are the primary obstacles in assessing the workload of ICU nurses. Additionally, the heightened demand for patient care services may impact the allocation of time for individual patient care by ICU nurses. After the implementation of the electronic medical record quality control system for ICU nurses, the time spent on quality control by these nurses decreased from 264 (195.5, 337.5) seconds to 62 (48, 76) seconds. The conventional manual quality control method is constrained by objective conditions and manpower, resulting in a substantial amount of time required for ICU quality control. When the beds are at full capacity, the ICU quality control nurse must review 23 medical records, which, according to our study, takes at least 6072 s. During this time, ICU nurses could administer nursing care to patients, including repositioning, back-patting, and rehabilitation exercises. Furthermore, the quality control results may be influenced by subjective factors of ICU quality control nurses, leading to potential deviations and challenges in ensuring the accuracy of nursing electronic medical records. In this study, an ICU nursing electronic medical record quality control system was developed using data platform integration technology. This system captures real-time data and offers automatic reminders based on predefined rules. Consequently, the time dedicated to manual inspection and omission evaluation by nurses was significantly reduced, resulting in improved work efficiency [13].

The findings in Table 1 indicate a significant reduction from 9 to 1.33% in the prevalence of inaccurate vital sign data in the ICU electronic medical records following the implementation of the ICU electronic medical record quality control system. Additionally, the rate of incomplete medication administration decreased from 3.33 to 1.67%, and the missed assessment rate decreased from 8 to 1.33%. These results may be attributed to the electronic medical record quality control system, which provided timely reminders for non-standard data and missed assessment items. Hence, omissions in nursing electronic medical records were identified and promptly rectified, thereby reducing the incidence of missed assessments. Additionally, the quality control system facilitated the detection and correction of false data, ensuring the authenticity and standardization of patient vital sign data.

Furthermore, the satisfaction of healthcare professionals with electronic medical records is a significant factor in determining the quality of health information systems [14]. In this study, the satisfaction score of ICU nurses with the quality control system for nursing EMRs was (105.73 ± 9.31), which showed a relatively high level. The results were similar to those of Zhang’s [15] study. Among the five dimensions of the evaluation scale, relatively high scores were obtained in user satisfaction, information quality, and net benefits. The analysis suggests that this may be related to the increased acceptance of nursing information technology by ICU nurses. The use of the nursing electronic medical record quality control system can not only improve the standardization of electronic medical records but also reduce the time spent on quality control. The interactive feedback between the system and the nurses can also promote the nurses’ awareness of the standardization of electronic medical records.

### Limitations

Nevertheless, the limitations of the current study should be acknowledged. Firstly, the study was conducted at a single tertiary care facility in China, which may limit the generalizability of the outcomes to other healthcare institutions. Secondly, while the sample size was adequate for this investigation, it remains relatively small when considering the broader context of ICU nursing environments, potentially affecting the external validity of the results. Additionally, potential sources of bias could not be excluded due to the constraints of the study design. For instance, the nurses’ adaptability to the new system and their individual skills may have influenced the assessment of satisfaction with the electronic medical record quality control system. Future multicenter studies should be conducted to validate the findings of this study and explore the potential impacts of implementing such a system in diverse healthcare environments.

## Conclusion

The findings of this study suggest that the ICU nursing electronic medical record quality control system has led to substantial improvements in the quality and reliability of EMRs. The reduction of false data on vital signs, instances of incomplete medication administration, and missing evaluations of assessment items demonstrates the system’s positive impact on nursing documentation practices. These improvements not only enhance the accuracy of patient records but also contribute to better patient care and safety within the ICU setting. The system has been well-received by ICU nurses.

## Data Availability

If reasonable requests can be made, the corresponding author will provide access to the datasets used and/or analyzed during this study.
